# Increasing substance P levels in serum and synovial tissues from patients with developmental dysplasia of the hip (DDH)

**DOI:** 10.1186/1471-2474-15-92

**Published:** 2014-03-19

**Authors:** Hui Wang, Xin-Feng Zheng, Xiang Zhang, Zheng Li, Chao Shen, Jun-Feng Zhu, Yi-Min Cui, Xiao-Dong Chen

**Affiliations:** 1 Department of Orthopaedic Surgery, Xinhua Hospital, Shanghai Jiaotong University School of Medicine, Kongjiang Road, No.1665, Yangpu District, Shanghai, China

**Keywords:** Substance P, DDH, Synovium, NK-1 receptor, Chronic pain, Osteoarthritis

## Abstract

**Background:**

The tachykininergic neurotransmitters have been proved to be involved in the inflammatory progress and chronic pain in series of disease. The present study was undertaken to evaluate the levels of substance P (SP) and its receptors NK-1 receptor (NK-1R) in both serum and synovial tissues of hip joint from patients with different stages of DDH, and to detect the possible correlation of serum SP levels with pain sensation and dysfunction of the hip joint.

**Methods:**

SP levels in serum and synovial tissues from patients with DDH and DDH combined with osteoarthritis (DDH&OA) group were compared through immunohistochemistry (IHC), ELISA, and 2-step acetic acid extraction method respectively. Expression of NK-1R in synovial tissues was compared through IHC, quantitive Real-Time PCR (QRT-PCR) and Western-Blot. The severities of pain sensation and the functional activities of hip joint were assessed by Visual analogue scale (VAS) and Harris hip score (HHS). Correlations of serum SP levels with VAS, HHS and erythrocyte sedimentation rate (ESR) were evaluated respectively in these groups.

**Results:**

Significantly elevated serum SP levels were detected in group of DDH and DDH&OA compared to that in normal group. IHC, QRT-PCR as well as tissue Elisa showed that SP levels in synovial tissue of DDH&OA group is stronger than that in DDH group. Serum SP levels in each group have no gender differences. The enhanced SP levels in synovial tissue mainly came from the segregation of peripheral nerve endings. Serum SP correlated with VAS and HHS in patients with DDH&OA (Male + Female). Serum SP correlated with HHS in patients with DDH (Male). Serum SP levels also correlated with erythrocyte sedimentation rate (ESR) in patients with DDH&OA (Male + Female). Up-regulated expression of NK-1R was also observed in synovial tissue of patients with DDH&OA compared to patients with DDH, through western-blot, IHC, and QRT-PCR.

**Conclusions:**

These findings indicated that the increasing SP levels in serum and synovial tissues, observed from patients with DDH to patients with DDH&OA, might associate with the loss of function and chronic pain sensation in hip joint. SP along with its receptors NK-1R might be involved in the progression of DDH into DDH&OA. In the future, inhibitors of SP as well as NK-1R may represent a novel pharmacotherapy target for pain relieving, inflammation alleviating and joint degeneration delaying for patients with DDH.

## Background

Developmental dysplasia of the hip (DDH), caused by developmental disorder of hip joint, results in shallow acetabulum, slacking joint capsule and subluxation of femoral head which always lead to chronic pain as well as joint dysfunction. The incidence rate of female and male with DDH is approximately 4–10:1 in different countries [1,2]. The pathogenesis of DDH includes: susceptibility to the X chromosome, genetic mutation, breech presentation, swaddling position, and mechanical factors of hip joint during acquired environment [3-5]. Besides, DDH is often accompanied by series of morphological and anatomic changes in hip joint, which bring about joint space narrowing, articular surface abrasion, secondary osteoarthritis, synovial hyperplasia, and cystic change in subchondral bone [6,7].

Pain in hip joint is a very common symptom for DDH, being related to both motion and rest. Furthermore, the degree of pain sensation is always associated with the progress of degeneration in hip joint according to clinical observations. However, so far, pathophysiologic mechanisms of pain in DDH are not yet clear. According to the known theories, femur acetabulum impingement, abrasion of articular surface and labrum injuries might all contribute to the occurrence of pain in hip joint [8,9].

Currently, the roles of tachykinin family and associated mediators played in pain and inflammation, have attracted increasing consensus in the field of molecular-biology on clinical disease [10,11]. Among these neuropeptides, substance P (SP) along with its specific receptors NK-1R were deemed as crucial factors in pain induction, which have been proved in both human and rat model of knee osteoarthritis and rheumatoid arthritis [12-14]. Besides, the up-regulation of SP and calcitonin gene-related peptide (CGRP) was observed in hip joint capsule, synovium and subchondral bone in patients with osteoarthritis [15,16]. On the other hand, being the member of tachykinin family, SP is mainly secreted by sensory neurons in both central and peripheral nerve systems [17], which indicated that these neurotransmitters probably play a vital role in joint pain mediation and transmission [18]. However, up to now, the investigation of occurrence and transmission of pain sensation in hip joint of DDH is still limited.

Therefore, this study was designed to detect the level of SP and its receptors NK-1R in both serum and synovium of hip joint in patients with DDH and DDH&OA. In addition, we also want to investigate the main source of SP in synovium, and the correlation of SP with chronic pain sensation and the function of hip joint in different stages of DDH.

## Methods

### Patients and materials

Clinical data and samples included in this study were obtained from patients underwent surgery treatment in our institution between March 2011 and December 2012. 45 hips in patients (ranges: 35–48 years, mean: 40.4 years) with chronic osteoarthritis combined with DDH (Crowe:I ~ III) were set to DDH&OA. Including criteria were osteoarthritis secondary to DDH, severe degenerative arthritis of grade 4 in the classification of Kellgren and Lawrence [19,20]. 56 patients with DDH (Crowe:I ~ III, ranges: 21–34 years, mean: 28.5 years) which defined as the hip having a sharp angle <45°and centre edge(CE) angle <20° [21,22] were included in this cohort. Severe pain sensation and dysfunction in hip joint were common performances for patients in both of these groups. Besides, 31 normal volunteers (ranges: 22–38 years, mean: 33 years) were also recruited in this study to serve as control group.

Synoviums in hip joint were obtained from patients with DDH&OA (n = 35), DDH (n = 20) separately, as shown in Table 1. In our study, patients with DDH&OA were included and served as the advanced stage of DDH, which help to investigate the content of SP and its receptors NK-1R in different stages of DDH. In order to decrease the effect from age and sex, we recruited the participants roughly the age and acquired the synovial tissues for research mainly from female patients.

**Table 1:** Data and clinical details of patients and normal people recruited in this study

	**DDH&OA**	**DDH**	**Normal**
Number	45	56	31
Ages (yrs)	40.4 ± 5.3 (35-48)	28.5 ± 6.4 (21-34)	33 ± 4.5 (22-38)
Female/Male	31/14	48/12	17/14
BMI (kg/m^2^)	23.5 ± 1.3	22.7 ± 0.8	22.2 ± 1.1
Durations (yrs)	3.8 ± 0.5	1.3 ± 0.4	—
CRP (mg/l)	8.4 ± 2.4*Δ	4.7 ± 1.5	2.4 ± 0.7
ESR (mm/1st h)	16.8 ± 4.6*	12.3 ± 3.6*	6.2 ± 1.6
Harris hip score	37 ± 6.4**#	75.7 ± 8.3*	96 ± 2.5
Visual analogue score	5.7 ± 2.4Δ	3.2 ± 1.8	—
Synovial sample	35/45	20/56	—
Blood sample	40/45	40/56	31

*BMI* Body mass index, *CRP* C-reactive protein, *ESR* Erythrocyte sedimentation rate. Data are given as mean ± standard deviation (SD). *P < 0.05 and **P < 0.01 compared to data in normal group. ΔP < 0.05 and #P < 0.01 compared to data in DDH group.

The diagnosis of DDH was based on impingement test as well as Sharp, CE angles in pelvic anteroposterior radiograph by chief physician (Dr Chen) in clinic. Clinical and laboratory data for the entire groups are presented in Table 1. Variables such as erythrocyte sedimentation rate (ESR) and C-reactive protein (CRP) was measured by clinical standard technology. Synovial tissues of hip joint in DDH&OA were acquired from the operation of Total hip joint arthroplasty (THA). Synovial samples in DDH were obtained during the operation of periacetabular osteotomy (PAO) and osteochondrocyte plasty (OCP) for patients with testify of opening joint capsule, which is characteristic in our department [23,24]. Samples of synovial in fossa acetabuli collected from operation were cut into pieces of 1 × 1cm^2^ and immerged into RNA Later (Ambion AM7021 USA) for 12 hs and then stored at −80°C for RNA and protein extraction.

### Criteria for exclusion

Patients recruited in this study had no secondary osteoarthritis (OA) as a consequence of inflammation, rheumatism, osteonecrosis, trauma, gout or tumor. Kidney or thyroid insufficiency was also an exclusion criterion. Drug therapies with OPIATES or NSAIDS were not allowed. Besides, patients with DDH subsequent by slipped epiphysis, traumatic osteoarthritis and those who had experienced orthopedic hip surgery were also excluded.

### Ethical justification

The study was performed based on the recommendations from the Declaration of Helsinki and was approved by the Ethical Committee of Shanghai Jiaotong University School Medicine Affiliate Xinhua Hospital. Samples prepared from operation were only for testing purposes, and were acknowledged, consented, and signed by patients.

## Quantification of substance P

### Serum test

Collected blood samples were allowed to clot for at least 30 min and then centrifuged at 3000 r/min for 15 min to obtain serum. Serum samples were then kept at −80°C before analysis. The concentration of SP in serum was determined by an enzyme linked immunosorbent assay (ELISA) kit (Cayman, catalog NO.583751, USA) according to the manufacturer’s instructions, which provides accurate measurements of SP with a working range of 1.5 to 500 pg/ml. It was verified by plenty of studies that the SP antibody used here recognizes the intact peptide and shows 100% specificity for SP, 93% for SP2-11, and 30% for SP7-11 and was without cross-reactivity with neurokinin A (2.7%) or neurokinin B (0.04%) [25,26].

### Synovium test

The concentration of SP in synovium was evaluated by the same ELISA Kit described above. SP levels in synovium were extracted by two-step 2% acetic-acid [27,28]. Briefly, 10-50 mg synovial samples were obtained from patients in separate groups as described in Table 1. We cut the tissue into fragments of 1-4 mm^3^. The first extraction was carried out by 1 ml 2% acetic-acid in 95°C for 15 mins. The supernatant regarded as SP from nerve endings was collected and then quickly frozen in −80°C. The second extraction was implemented by 1 ml 2% acetic-acid in 95°C for 45 mins. Supernatant was extracted and deemed as SP from non-nerve tissue or cells. The levels of SP were normalized to the weight of the tissue samples and expressed as pg/mg.

### Assessment of HHS and VAS score

Assessment of hip joint mobility was performed using Harris hip score (HHS) by Dr Zhu. Along with HHS score, a visual analogue scale (VAS) was also applied for the assessment of pain severity of patients in separate group. In order to determine VAS score, a 100-mm horizontal line without scaling was designed in which 0 was marked as “no pain in hip” and 10 was marked as “unbearable pain”. Patients were then instructed to place a vertical mark reflecting their soreness severity. Scores of both HHS and VAS in different groups were shown in Table 1.

### RNA isolation and quantitative real-time PCR (QRT-PCR)

Synovial tissues from fossa acetabuli were obtained during operation of total hip arthroplasty (THA) or Bernese periacetabular osteotomy (PAO) as previously described from patients of 20 with DDH, 35 with DDH&OA. The tissues were dissected into small pieces of 80 mg and immediately frozen in liquid nitrogen. Frozen tissues (100 mg) were grinded by mortars into granular powder and was dissolved in 2 ml volumes of Trizol reagent kit (Takara, Code: D9108B, Japan). cDNA was synthesized from 2 mg total RNA through reverse transcription using a TaKaRa RNA PCR Kit (TaKaRa, DRR057A, Japan) according to the manufacturer’s protocol. The sequences for primers (TaKaRa, Biotechnology Co. Ltd, Japan) used to amplify mRNA were as follows: TAC1R: F: 5′-GTCGTGTGCATGATCGAATGG-3′, R: 5′-TTGCTCGTGGTAGCGGTCAG-3′; TAC1: F: 5′-GGTACGACAGCGACCAGATCA-3′, R: 5′-CCCGTTTGCCCATTAATCCA-3′. Real-time PCR was carried out using 200 ng of cDNA and PrimeScript® RT-PCR Kit (Takara, DRR064A, Japan) in 96-well plates in a ABI 7500 Sequence Detection System (ABI, Co. Ltd, USA) according to the manufacturer’s instructions. Relative quantification of each gene was normalized to GAPDH. We used the 2^-ΔΔCt^ (cycle threshold) method to calculate relative gene expression levels as previously described [29]. Results are presented as fold changes in gene expression normalized to GAPDH and relative to control conditions (DDH group). Data of each sample was displayed on triplicate wells during every PCR amplification circulation. Analysis of the results is based on at least three individual experiments.

### Immunohistochemistry and assessment

The synovial samples were dehydrated in a graded series of ethanol and embedded in paraffin for immunohistochemistry. The tissues were cut into sections measuring 5 um and were mounted on anti-off slides. Sections from each group underwent citrate buffer antigen retrieval. After incubated overnight with the primary antibody NK-1R (1:1000) (Abcam, ab75516, UK) and substance P(1:1000) (Abcam, ab14184, UK), slides were rinsed in phosphate buffer saline (3×5 minutes) and incubated with biotinylated secondary antibody (Dako, Ely, UK). Finally, streptavidin-biotin complex (1:2000) (Amersham Life Science Inc, USA) was used for the visualization of the immunoreactions. From every synovium (8 per group), two sections were acquired at different depths and eight microscopic fields from each section were photographed and analyzed. Thus, in every synovial sample of separate group, 16 microscopic fields were collected. Images of the microscopic fields were captured by a camera (DEI 750, Optronics Engineering, USA) attached to the microscope. The immune activity was measured as the area in mm^2^ using the software (Image-Pro plus 6.0, USA) as previously described [30]. The results were expressed as the positive staining area (mm^2^) in relation to the total area of each microscopic field.

### Western-blot analysis

Synovial samples of different groups (150 mg/sample) were grounded into powder with liquid nitrogen. Total protein was extracted using a Western & IP Cell Lysis Kit (Beyotime, Shanghai, China). The concentration of protein in the cell lysate was determined using a microplate reader (Thermo, Finland) according to the manufacturer’s protocol. For each sample, the whole cell extracts equivalent to 40 mg total protein which were loaded into a 10% SDS–polyacrylamide gel, separated by electrophoresis and electrotransferred to a polyvinylidene difluoride (PVDF) membrane. Non-specific binding was blocked by incubating the PVDF membrane with 10 mM TBS, 1.0% Tween, and 10% dehydrated skimmed milk. Following the blocking procedure, the membranes were incubated overnight with antibodies against NK-1R (1:1000) (Abcam, ab75516, UK) in 4°C. The performance of immunoblotting on the same membranes using antibodies against GAPDH (Beyotime, Shanghai, China) was used as a loading control to assess the group differences. Gray-value of respective well in image strip was analyzed by software (Image J, NIH, USA) and normalized to value of GAPDH. Data of each group was acquired by three times of experiments at least.

## Results

Samples and blood index acquired from separate groups were shown in Table 1. Mean ages were 28.5 and 40.4 years in groups of DDH and DDH&OA respectively. Significant differences in serum CRP, ESR as well as VAS and HHS were observed in comparisons among DDH, DDH&OA and Control group.

### Change of SP levels in serum and synovium

#### SP in serum

**Figure 1:**
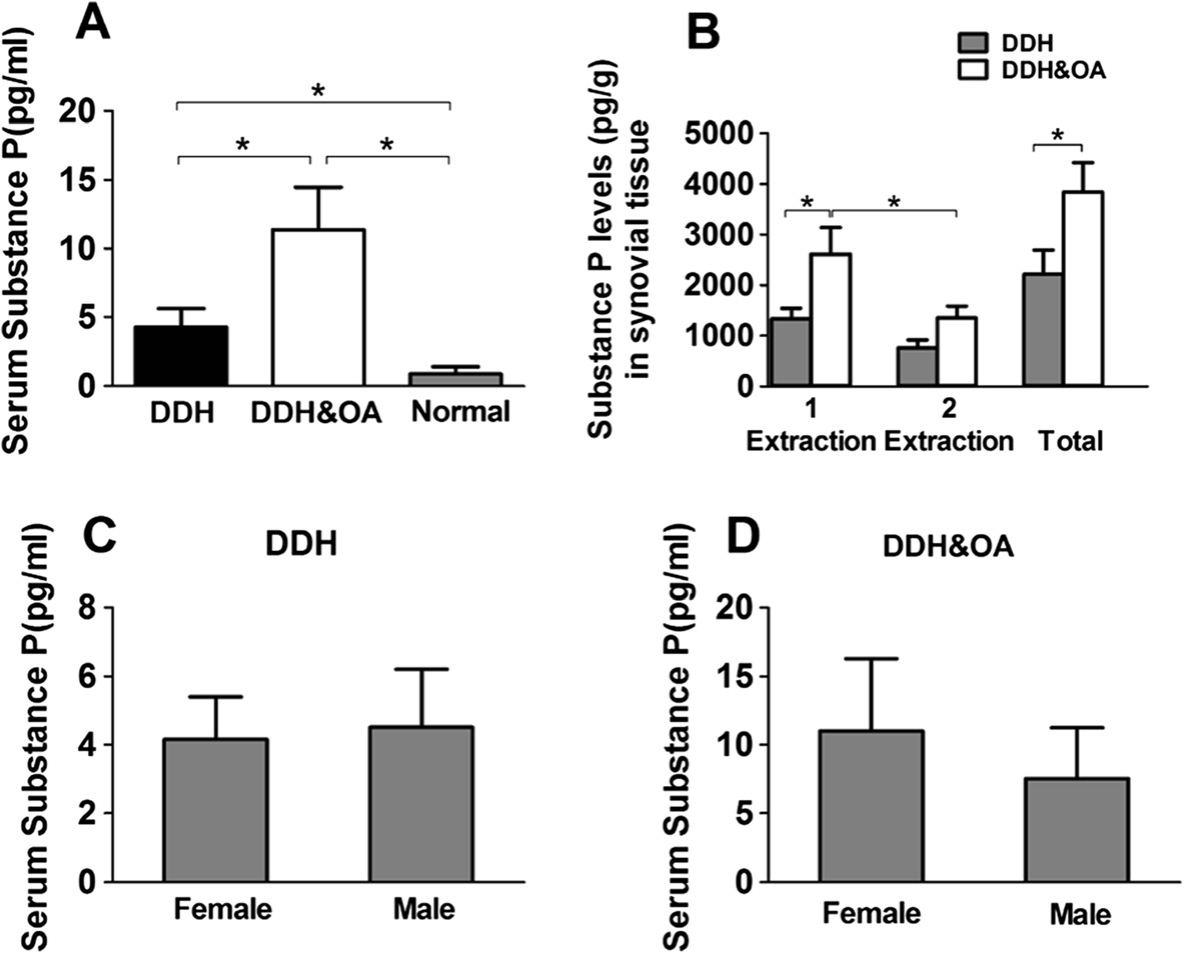
**Levels of substance P in both serum and synovium from patients among different groups. (A)** Contents of SP in serum by an ELISA assay. Serum samples were obtained from patients with DDH (n = 40), DDH&OA (n = 40) and normal people (n = 31) recruited in this research. **(B)** Concentration of SP levels in synovial of fossa acetabuli from patients in separate groups. 1 extraction stands for SP secreted from free nerve endings in synovial tissue, whereas 2 extraction means SP generated from non-neuronal cells (*P < 0.05). **(C)-(D)** Gender differences of serum SP levels was investigated in each group (*P < 0.05).

Concentrations of serum SP in patients from different groups were presented in (Figure 1A, C-D). From (Figure 1A), significantly higher levels of SP could be observed in group of DDH and DDH&OA compared to control group (P < 0.05). Furthermore, SP levels in DDH&OA group show obvious difference compared to that in DDH group. Additionally, we did not obtain significant differences of serum SP levels between male and female in each group (Figure 1C-D).

#### SP in synovium

To identify the concentration of SP in synovial tissue of hip joint, and to the possible sources of SP, we adopted the two-step extraction method which has been described above. According to the outcome of ELISA (Figure 1B), SP is found both in the first and second extraction which stands for free nerve endings (neuronal) and non-neuronal tissues respectively. According to the remarkable difference of SP levels between the first and second extraction in DDH&OA (Figure 1B), we could speculate that the production of SP in synovium was mainly secreted from peripheral neuron which present as the first extraction. Moreover, from the first and total extraction from synovium, we could get significantly higher SP levels in DDH&OA than that in DDH.

### Correlation of SP in serum with VAS, HHS and ESR in patients among different groups

As expected, patients in DDH&OA group had obvious higher score in VAS (P < 0.05) but lower score in HHS (P < 0.01) compared to that in DDH group. In the correlation detection (Table 2), significant positive correlations were found in group of DDH&OA (Female + Male): SP/HHS: r = −0.496, P < 0.01; SP/VAS: r = 0.512, P < 0.01. DDH&OA (Male): SP/HHS: r = −0.694, P < 0.01. DDH (Male): SP/HHS: r = −0.82, P < 0.01. Furthermore, we also observed a strong correlation of SP in serum with ESR(r = 0.508, p < 0.01) in patient with DDH&OA (Female + Male).

**Table 2:** Correlation test of serum SP levels with HHS, VAS and ESR in patients with DDH and DDH&OA

**Correlation of serum substance P levels**								
**Group**	**Sex**	**N**	**With HHS**		**With VAS**		**With ESR**	
			**R**	**P**	**R**	**P**	**R**	**P**
DDH								
	F	30	−0.344	0.063	0.216	0.251	0.086	0.652
	M	10	−0.820	<0.01*	0.591	0.072	0.407	0.243
	F + M	40	−0.219	0.175	0.349	0.027	0.156	0.336
DDH&OA								
	F	27	−0.384	0.048	0.355	0.069	0.497	0.083
	M	13	−0.694	<0.01*	0.428	0.144	0.437	0.135
	F + M	40	−0.496	<0.01*	0.512	<0.01*	0.508	<0.01*

*F* female, *M* male, *N* number, *R* correlation coefficient. Spearman correlation test was implemented to investigate the possible correlations in separate groups. A two sides *P < 0.01 means significant correlation.

### NK-1R is up-regulated in synovial tissue of patients with DDH&OA compared to DDH

**Figure 2:**
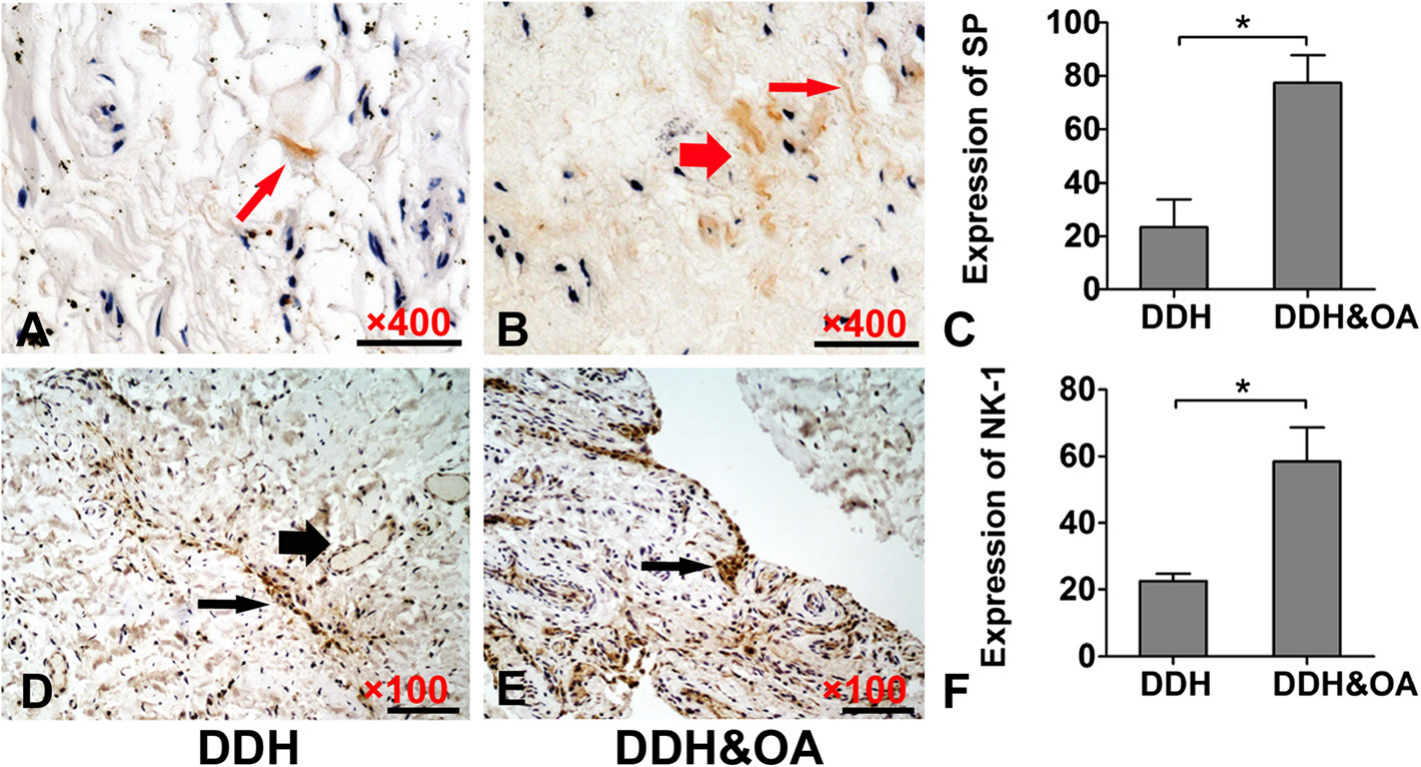
**IHC staining and gray value analysis of substance P and NK-1R in human synovial of fossa acetabuli.** (**A**-**B**: IHC of SP), (**D**-**E**: IHC of NK-1R). Representative images are from patients with DDH **(A and D)**; DDH&OA **(B and E)**. **(C and F)** stands for gray value analysis of the IHC staining (*P < 0.05). Scale bars: **(A-B)** 400 times magnified; **(D-E)**: 100 times magnified. Thin arrows in **(A-B)** represent positive staining of nerve fibers-like SP. Thick arrows in **(B)** indicate nerve bundles-like SP. Thin arrows in **(D-E)** display fibers-like staining of NK-1R. Thick arrows in **(D)** show perivascular located staining of NK-1R.

**Figure 3:**
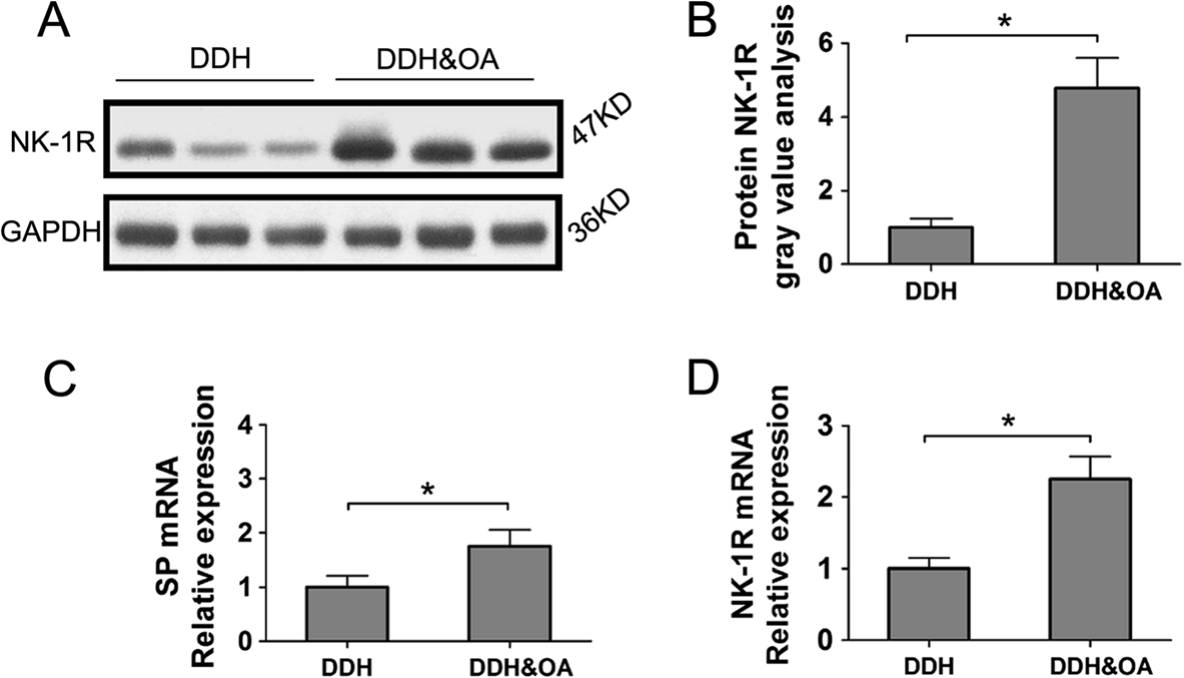
**Protein and mRNA expression levels of NK-1R and SP in synovial tissue of fossa acetabuli from patients in DDH and DDH&OA groups. (A)**, **(B)**: Western-blot analysis for NK-1R production levels in DDH and DDH&OA groups. **(C)**, **(D)**: Relative mRNA expression of SP and NK-1 in synovial tissues of separate groups. Relative gene expression was normalized to the housekeeping gene GAPDH (*P < 0.05).

According to (Figure 2D-F), we can observe significantly intensive staining of NK-1R in DDH&OA compared to that in DDH. Besides, staining of NK-1R mainly located in synovial cells as well as capillary vessel wall cells. In addition, western-blot and gray analysis reflected obviously up-regulated NK-1R levels in DDH&OA group compared to that in DDH group (Figure 3A-B). Similarly, through QRT-PCR, we also witnessed analogical tendency about the mRNA expression of NK-1R in these groups (Figure 3D).

### SP expression in synovial tissue

IHC staining as well as QRT-PCR were implemented to detect the expression of SP in synovium of hip joint. According to IHC staining and gray value analysis (Figure 2A-C), positive staining of SP was mainly observed in silk-like and nerve bundle-like nerve endings in synovium. And we can easily get stronger staining of SP in DDH&OA compared to DDH group. So it is with the comparison of mRNA expression of SP from synovial tissue between these groups (Figure 3C).

## Discussion

It is extensively accepted that, in advanced cases of DDH, which frequently combined with severe degeneration and osteoarthritis, severe pain sensation and dysfunction in hip joint would always be the primary complaint [31,32]. Current studies have focused mainly on the members of tachykinin family as well as its roles in the pain creation of osteoarthritis. Besides, the relationship between neuropeptides and cytokines in joint fluid and serum of patients with OA or RA were also studied [33,34]. Moreover, BN/GRP and SP in both serum and synovial fluid were found correlated with inflammation index such as CRP and ESR in patients with RA in hip joint [35]. These observations indicated that, as a typical nerve tachykinin, SP as well as some of the other neuropeptides might associated with inflammation and degeneration in hip joint and may also have some relationships with the occurrence of pain sensation.

In our study, the level of nerve tachykinin SP is up-regulated in both serum and synovial tissue from patients with DDH&OA compared to that in patients with DDH. Through serum test, we also observed some positive relationship between SP and ESR. These findings suggest that SP might reflect somatic pain sensation to some extend and associate with the severity of inflammation. As we know, SP along with other neuropeptides can stimulate the release of inflammatory mediators such as IL-1, IL-6, IL-8, and TNF-α from both blood monocytes as well as fibroblast-like synoviocyte (FLS) in human body [36-38]. Moreover, SP may favor the generation of memory Th17 cells by inducing IL-1β, IL-23, TNF-like 1A expression by monocytes [39]. These mediators on the one hand could act as chemoattractant molecule for further leucocyte migration. On the other hand, they might also directly bring about inflammatory response of the target cells through corresponding receptors and up-regulate the production of PGE2 from inflammatory cells which will advance the inflammation and pain [40].

In our study, NK-1R, the specific receptors of SP, revealed strong and visible immune-staining tendency in synovial tissue from varied stages of DDH group. Previous studies have proved that inflammatory reactions in response to an immune irritation are strengthened in neuropathic animals and are partly mediated by NK-1R [41-43]. Therefore, in this study, the up-regulated expression of NK-1R probably reflected a feedback, inducing series of intracellular signaling mechanisms to fit the negative stimuli outside. Also, researches demonstrated the validity of NK-1R antagonists in relieving cutaneous orofacial inflammatory pain and reducing hyperalgesia and cartilage destruction in the inflammatory joint in rats with adjuvant-induced arthritis [44,45]. Herein, we might hold the hypothesis that NK-1R antagonists could have therapeutic value in the treatment of pathological changes and the relief of arthritis evolvement in patients with DDH.

We know that SP stems from both neuronal and non-neuronal cells such as fibroblasts and some immune cells within the synovial tissue. According to the comparisons from tissue ELISA [Figure 1B], we speculate that SP in synovial tissue might mostly derived from segregation of peripheral free nerve endings, which coincided with that of previous research [46,47]. As neuropeptides were mainly secreted from DRG and transmitted through peripheral free nerve fibers, our findings indicated that more nerve fibers might invade into synovial tissue with the progress from DDH to DDH&OA, which probably involved in the upgrading of inflammation and pain sensation.

Several limitations also existed in this study. Firstly, it is impossible to obtain perfectly normal synovial specimens according to medical ethics requirement. Secondly, the age of patients in these groups was as close as possible. In our research, the mean age of DDH&OA group was 40.4 years, while the average age of patients with DDH was 28.3 years. We know that SP levels in tissue could change with ages [48,49]. Hence, doubtlessly, age differences between groups could affect the results. However, previous research has proved that SP levels in both animals and human may decrease with age [50,51]. In our study, patients in group of DDH&OA who were older than group of DDH represented higher levels of SP in both serum and tissue, which further certified that SP levels increase conspicuously in DDH&OA group compared to DDH group.

## Conclusions

In summary, our data firstly demonstrate that SP levels increased in serum of patients with DDH. Further increase of SP levels was observed in serum and synovial tissue, along with the progression from DDH to DDH&OA. This increase might cope with the dysfunction of hip joint as well as the upgrade of pain sensation. These findings together with previously published investigation, suggest that SP might associate with the creation of chronic pain and the inflammation progress of hip joint in DDH. SP along with its receptors NK-1R might become a promising therapy target for future drug research in relieving the progress of pain sensation and arthritis severities in DDH. However, how SP involved in the progression of arthritis and its possible bio-mechanisms performed on the degeneration of cartilage and subchondral bone in DDH still need further exploration.

## Abbreviations

DDH: Developmental dysplasia of the hip; DDH&OA: DDH combined with osteoarthritis; SP: Substance P; NK-1R: Neurokinin 1 Receptor; VAS: Visual analogue scale; ANOVA: Analysis of variance; HHS: Harris hip score; IHC: Immunohistochemistry; FLS: Fibroblast-Like synoviocyte; ESR: Erythrocyte sedimentation rate; PVDF: Polyvinylidene difluoride; CGRP: Calcitonin gene-related peptide; CRP: C-reactive protein; PAO: Periacetabular osteotomy; OCP: Osteochondrocyte plasty; THA: Total hip arthroplasty; SD: Standard deviation; GAPDH: Glyceraldehydes phosphate dehydrogenase; BMI: Body mass index.

## Competing interests

The authors declare that they have no competing interests.

## Authors’ contributions

HW and XFZ participated in the design of the study, manuscript preparation, data statistical analysis, and revision. XZ and ZL carried out the molecular biology studies. CS participated in manuscript preparation and revision. JFZ helped to assess HHS and VAS of patients involved in this study. YMC was involved in collecting serum and synovial samples in sickroom and operation room. XDC is responsible for study design, financial support, and general supervision of the research group. All authors read and approved the final manuscript.

## Pre-publication history

The pre-publication history for this paper can be accessed here:

http://www.biomedcentral.com/1471-2474/15/92/prepub
